# The secretion of von Willebrand factor from endothelial cells; an increasingly complicated story

**DOI:** 10.1111/jth.12225

**Published:** 2013-06-30

**Authors:** T Nightingale, D Cutler

**Affiliations:** * MRC Laboratory for Molecular Cell Biology, University College LondonLondon, UK

**Keywords:** endothelial cells, exocytosis, hemostasis, Rab GTPase, von Willebrand factor, Weibel–Palade bodies

## Abstract

von Willebrand factor (VWF) plays key roles in both primary and secondary hemostasis by capturing platelets and chaperoning clotting factor VIII, respectively. It is stored within the Weibel–Palade bodies (WPBs) of endothelial cells as a highly prothrombotic protein, and its release is thus necessarily under tight control. Regulating the secretion of VWF involves multiple layers of cellular machinery that act together at different stages, leading to the exocytic fusion of WPBs with the plasma membrane and the consequent release of VWF. This review aims to provide a snapshot of the current understanding of those components, in particular the members of the Rab family, acting in the increasingly complex story of VWF secretion.

## Introduction

Endothelial von Willebrand factor (VWF) is well understood to play a key role in hemostasis, acting directly to recruit platelets to sites of vascular damage, and indirectly by chaperoning clotting factor VIII to reduce its degradation and clearance. For VWF to be effective, it must be secreted in a timely manner, at sufficient levels, and in an appropriate structural format. These all rely on the proper functioning of its carrier organelles, Weibel–Palade bodies (WPBs). WPBs provide an environment in which the multimerization and folding of VWF can occur, they control the quantum of VWF release via their size, and they are actively involved in delivering VWF into the plasma. The secretion of VWF is thus a complex process orchestrated by intraorganellar events on the one hand, and cytoplasmic machinery on the other.

**Fig 1:**
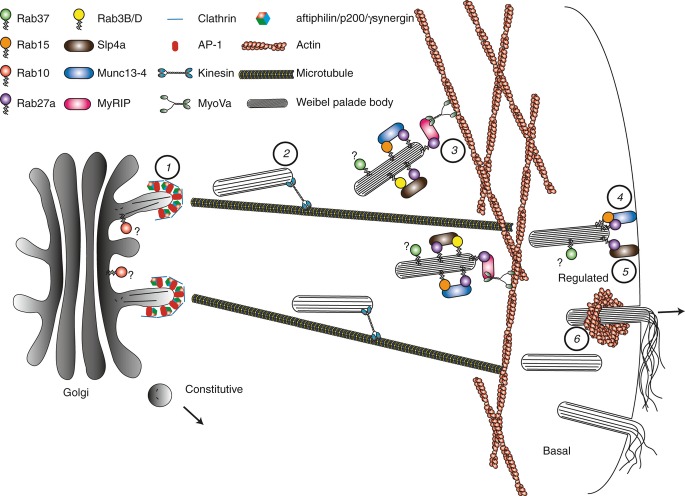
Formation and function of Weibel–Palade bodies (WPBs): focus on Rabs and their effectors. (1) The biogenesis of WPBs begins at the trans-Golgi network, where tubules of von Willebrand factor (VWF) are inserted into the nascent storage organelle. This process is dependent on the presence of a clathrin coat and the adaptor protein AP-1. Inhibition of either of these proteins results in a failure to form WPBs and constitutive secretion of VWF (normally negligible). AP-1 also recruits the tripartite complex of aftipilin, p200, and γ-synergin, which is required (presumably by the recruitment of as yet unknown proteins) for the regulated release of VWF. Rab10 is also required for the rapid regulated release of at least some VWF by a currently unknown mechanism. (2) Immature WPBs are trafficked by an as yet uncharacterized kinesin(s) to the periphery of the cell along microtubules. (3) Immature WPBs can become anchored on F-actin by a tripartite complex consisting of Rab27a, myosin and Rab27a-interacting protein (MyRIP), and myosin Va (MyoVa), and this allows peripheral localization and maturation (further multimerization of VWF, condensation of tubules, and an increase in WPB length) of the WPBs for later regulated release; alternatively, the immature WPBs are released basally at the cell surface, resulting in relatively short strings of VWF. WPBs also recruit Rab3B/Rab3D and the Rab27a/Rab3 effector Slp4a (granuphilin), as well as Rab15 and the Rab27a/Rab15 effector Munc13-4. (4) Following secretagogue stimulation, anchoring on actin is lost, and a Rab27a-dependent, Rab15-dependent and Munc13-4-dependent step at the cell surface is required for exocytosis to occur (potentially via an interaction with Doc2α). (5) A Rab27a-dependent and Slp4a-dependent docking step is necessary for release (potentially via an interaction with syntaxin or Munc18-1/Munc18-2). (6) VWF is released as high molecular weight strings at the cell surface in a manner that is expedited by the contraction of a ring of actin.

WPBs were first seen by Ewald Weibel 1, then working with George Palade at Rockefeller University in New York on St Valentine's day 1962, and their initial characterization during the early years has recently been well described by their discoverer 2,3. Their remarkable shape might have made them a prime target for cell biologists, but, in fact, our understanding of these organelles has lagged behind that of other, much less morphologically striking, anterograde carriers, such as chromaffin or insulin granules. Recently, reviews of their structure and function have become more frequent as interest in their unique properties has increased 4–6. Furthermore, the number of other components associated with WPBs is increasing in parallel with additional studies 7 (and references therein). A brief overview of WPB formation and function will be provided here for background, but this review will focus on the most recently active areas of research, in particular the machinery of exocytosis, the modulation of exocytosis, and the roles of Rabs and their effectors in these events. A schematic of the role of Rabs in VWF secretion is shown in Fig.1, and summaries of the known WPB Rabs and their effectors are shown in Tables1 and 2, respectively.

**Table 1:** Rab proteins recruited to Weibel–Palade bodies (WPBs)

Rab	Determination on WPBs	Method	siRNA effect on VWF release	Comment	Reference
Rab27a	Endogenous expression and overexpression	Western blot, RT-PCR, and IF	Increase or decrease	–	26,34,47,50
Rab27b	Overexpression	RT-PCR	Decrease	Level of expression very low	47
Rab3A	Overexpression	RT-PCR	Decrease	–	47
Rab3B	Endogenous expression and overexpression	Western blot, RT-PCR, and IF	No effect	–	47,50
Rab3D	Endogenous expression and overexpression	Western blot, RT-PCR, and IF	Decrease or no effect	Endogenous Rab3D weakly detectable in 5% of cells	47,50,52
Rab15	Overexpression	RT-PCR	Decrease	–	47
Rab33a	Overexpression	RT-PCR	No effect	–	47
Rab37	Overexpression	RT-PCR	No effect	Low expression (cycle time 39.74)	47

IF, Immunofluorescence microscopy; siRNA, small interfering RNA; VWF, von Willebrand factor.

**Table 2:** Rab effector proteins recruited to Weibel–Palade bodies (WPBs)

Rab effector	Determination on WPB	Method	siRNA effect on VWF release	Comment	Reference
MyRIP (Slac2c)	Endogenous expression and overexpression	Western blot, RT-PCR, and IF	Increase	Binds Rab27a* and MyoVa*/MyoVIIa	19,26,33,50,80
Slp4a (granuphilin)	Endogenous expression and overexpression	Western blot, RT-PCR, and IF	Decrease	Binds Rab27a* (GTP-bound and GDP-bound), Rab3A-D*, Rab8, syntaxin 1a, syntaxin 2, syntaxin 3, Munc18-1, and Munc18-2	50,55,56,81
Munc13-4	Overexpression	Western blot	Decrease	Binds Rab27a/b*, Rab15*, and Doc2α.	47,82,83
MyoVa	Endogenous expression and overexpression	Western blot, RT-PCR, and IF	Decrease	Binds MyRIP*, Slp4a, Rab8, Rab3, Rab10, and melanophilin	19,59,84–86

IF, Immunofluorescence microscopy; MyoVa, myosin Va; MyoVIIa, myosin VIIa; MyRIP, myosin and Rab27a-interacting protein; siRNA, small interfering RNA; VWF, von Willebrand factor.

* Interaction that has been shown to occur in endothelial cells.

## Overview of VWF secretion

Two parallel interdependent activities underpin the release of VWF. One is the biosynthesis of the remarkable structure that is the mature VWF protein assembly; multimerized, coiled, and ready to be released. The other is the formation and maturation of a cigar-shaped carrier, which is enormous by comparison with other secretory organelles. Without VWF, WPBs cannot form. Without the specialized carrier, VWF cannot attain its functionally active structure.

### Biosynthesis of VWF

The VWF gene encodes a 2813 amino acid protein with a 22 amino acid signal peptide and a 741 amino acid propeptide. Initially forming a dimer within the endoplasmic reticulum via C-terminal disulfide bonds, it travels along the anterograde pathway into the Golgi apparatus, where interchain disulfide bonds are formed, the propeptide is cleaved, and the remodeling of the multimerizing protein into a proteinacious tubule commences. VWF is incorporated into an immature carrier at the trans-Golgi network (TGN), where the earliest-forming VWF tubules can be seen by electron microscopy (EM) 8. The immature WPB then carries the VWF towards the cell periphery, and, during its subsequent maturation, the VWF continues multimerizing and forming tubules 9. At exocytosis, very high molecular weight VWF is released as long platelet-catching strings 10,11 that are particularly effective in primary hemostasis. Our rapidly improving understanding of the structural aspects of multimerization and tubulation has been recently reviewed elsewhere 4,12.

### Formation of WPBs

The formation of WPBs at the TGN has been evident from the earliest EM investigations, even before it was realized that the tubular content used as a marker of that biogenesis reflected the presence of VWF 13. This initial formation is driven by the tubulation of VWF, as heterologous expression of VWF in certain other cell types drives cigar-shaped WPB formation 14, and clear distortion of the surrounding membrane accompanies even initial VWF tubulation 8. Interestingly, a cytoplasmic supporting structure is also required; a clathrin coat is clearly present on the cytoplasmic face of forming WPBs 15.

In the absence of the heterotetrameric clathrin adaptor AP-1, a failure to recruit clathrin leads to the formation of small VWF-containing carriers that are not capable of supporting tubulation of VWF, and that do not respond to secretagogue 15. AP-1 also plays a more active role in the formation of functional WPBs, because, in addition to recruitment of the structural protein clathrin, it also recruits the trimeric complex of p200, aftiphilin, and γ-synergin, a poorly understood complex that is essential for the targeting of WPBs into the regulated secretory pathway 16. In their absence, AP-1 can still recruit clathrin, and VWF is still organized into WPBs of identical morphology to controls, but there is a loss of regulated secretion and a concomitant rise in basal secretion (see below). The exact mechanism of aftiphilin–p200–γ-synergin action is enigmatic, but the localization of this complex on immature perinuclear WPBs suggests the early recruitment of a factor required for regulated release or (less likely) of an inhibitor that blocks basal release. AP-1 therefore coordinates the targeting and formation of WPBs. In the absence of controlled delivery to the blood (aftiphilin–γ-synergin-dependent), formation of the most highly prothrombotic forms of VWF (clathrin-dependent) is also prevented. Thus, the most highly prothrombotic forms of VWF cannot be made unless the secretion of those organelles is regulated. Coupling of the two processes via their mutual dependence on AP-1 thus provides a useful checkpoint (Fig.1).

After becoming independent of the TGN, WPBs are redistributed via interactions with both microtubules (via dynein and an as yet undefined kinesin[s]) and actin filaments (via myosin Va [MyoVa]) in such a way as to shift the overall distribution of the cell WPB population to the periphery 17–20. In parallel, recruitment of further components, including the integral membrane protein CD63 21,22 and the Rabs that play a major role in WPB functioning (see below), occurs, as well as some remodeling of the WPB limiting membrane and the probable removal of material. This remodeling process is especially poorly understood, and is currently represented only by EM images of clathrin-coated buds on WPBs 8. Neither specific cargoes nor the adaptor(s) involved have yet been identified.

Secretion of VWF can take place by one of three pathways: regulated, secretagogue-stimulated release of VWF from WPBs; basal, secretagogue-independent release of VWF from WPBs; or constitutive, secretagogue-independent release of VWF from non-WPB anterograde carriers. Initially, most secretion of VWF from endothelial cells was thought to occur constitutively 23. However, a study of the multimeric state of endothelial releasates by Tsai *et al.* 24 challenged this view, and suggested that the majority of VWF was secreted by the regulated secretory pathway. Most recently, Giblin *et al.* 25 revisited the issue, and concluded that most of the VWF released in the absence of secretagogue stimulation is from WPBs, i.e. by basal secretion. Release of VWF from constitutive carriers is therefore only apparent when a component of the cytoplasmic machinery such as AP-1 is ablated 15.

Post-scission, the immature WPB will either undergo exocytosis basally or become anchored to F-actin, where maturation continues before exocytosis 26. Regulated exocytosis will follow activation of the endothelial cells by any of a large number of different secretagogues 27.

## Exocytosis of WPB; mechanisms and machinery

Bringing an organelle to the plasma membrane for subsequent exocytosis is a multistep process, involving sequential stages that may include tethering, docking, priming, and fusion 28. In the case of WPB exocytosis, much is not yet understood, including which of the above stages may exist in the endothelial regulated secretory system (Fig.1).

### Peripheral anchoring of WPBs to allow maturation

There is an absolute physiologic requirement for the release of high molecular weight VWF multimers, as an absence of just the very highest molecular weight forms results in a bleeding diathesis (such as observed in type 2A and type 2B von Willebrand disease) 29. Given that multimerization continues in WPBs after their scission from the TGN 9, this places particular importance on this maturation step. This pre-exocytic stage is supported by Rab27A, a GTPase that is often associated with lysosome-related regulated secretory organelles, where it generally acts via its effectors to link organelles to cortical actin filaments 30–33. Rab27A was the first WPB Rab to be identified, and Hannah *et al*. showed that this Rab is not only recruited to WPBs in endothelial cells, but also recruited to the pseudo-WPBs induced by the heterologous expression of VWF in HEK293 cells. These data show that Rab27A is recruited by a remarkable content-driven, maturation-dependent mechanism that is independent of cell type 34.

Subsequent work by Nightingale *et al*., using small interfering RNA (siRNA)-mediated suppression of Rab27A, revealed a number of related phenotypes. First, depletion in human umbilical vein endothelial cells (HUVECs) over a period of 6 days led to a redistribution of WPBs from a peripheral to a more perinuclear localization. Second, this redistribution was accompanied by increases in both basal, phorbol-12-myristate-13-acetate (PMA)-evoked and histamine-evoked release. Third, the VWF released from Rab27a-deficient cells is less highly multimerized than that released from controls, and gives rise to shorter VWF strings under flow (incidentally demonstrating a link between degree of multimerization and length of strings). These data suggest that Rab27A acts to modulate the maturation state of the released VWF in a manner that is dependent on the intracellular distribution of WPBs; Rab27A thus acts to anchor the WPBs to filamentous actin and inhibit release to allow appropriate maturation, and full multimerization to be completed. Secretagogue activation of the endothelial cell leads to release from anchorage, and fusion with the plasma membrane, leading to secretion of the VWF 26.

All Rabs act through their effectors. Rab27A has 11 reported effectors, and Nightingale *et al*. found that several of these were expressed in HUVECs, including myosin and Rab27a-interacting protein (MyRIP), granuphilin (Slp4a), Slp3, Noc2, Munc13-4, and Slp2a. They analyzed the role of MyRIP, the most highly expressed of these, in HUVECs. They found that Rab27A is recruited to maturing WPBs, that this is followed by recruitment of MyRIP, and that the acquisition of MyRIP by WPBs is associated with peripheral distribution. The effects of siRNA suppression of MyRIP were similar to those of siRNA suppression of Rab27a, i.e. an increase in PMA-evoked release of VWF, and a marked loss of the more peripherally located WPBs. The secretion phenotype found following suppression of MyRIP was less marked than that seen following suppression of Rab27a, suggesting potential additional roles for other effector proteins 26. Subsequently, Pulido *et al.* 19 showed that the actin-binding protein MyoVa binds to MyRIP, completing a tripartite link between F-actin and the WPB.

### Modes of exocytic fusion events

The secretion of material from granules can occur in several modes. In addition to simple exocytosis of secretory carriers fusing individually with the plasma membrane, in some cells compound multivesicular or cumulative fusion has been reported 35. The former refers to granules fusing with each other and then the plasma membrane, as in mast cells, where massive simultaneous release of all secretory cargoes occurs 36. The latter is a variant where, after initial fusion of a granule with the plasma membrane, subsequent granules fuse to that same structure. Eosinophils use a mixture of both of these strategies to produce a massive, but focused, release of cytotoxic proteins 37.

Valentijn *et al*. used EM analyses of activated endothelial cells to suggest that WPBs exocytose via multivesicular compound exocytosis. They describe a phenomenon whereby there is intracellular homotypic fusion of WPBs into a super-sized collapsed granule to produce a structure that they name a ‘secretory pod’ 38. This then initially fuses to the plasma membrane via a thin tubule, similar to that seen by Rosenthal *et al*. in one cryo-EM image, where it is apparently connecting a single cigar-shaped WPB to the plasma membrane 39. The initial description by Valentijn *et al*. leaves many unanswered questions, such as whether this is an obligatory route for release of VWF – which is improbable, as the movies from other laboratories 40–44 overwhelmingly show exocytosis from single granules – or a minor variant. If the latter is the case, then under what physiologic circumstances might it be employed by endothelial cells, and what would be the advantages and disadvantages for the cell? Finally, the data currently available do not yet rule out cumulative compound exocytosis as an additional possibility for WPB fusion.

Even the fusion event itself is not simple in the case of WPBs. A detailed analysis of WPB fusion by Babich *et al.* 41 revealed that this is not an all or nothing event. In common with other secretory systems 45, the exocytic pore can remain open after initial fusion until all of the content has been released (full fusion), or can reclose (kiss and run). Babich found a stimulatory regime for endothelial cells that drives WPBs into a state that they colorfully describe as a long lingering kiss. This allows for the effective release of smaller molecules, such as cytokines, but retains the VWF within the collapsed WPB (after fusion, the open pore leads to a rise in intra-WPB pH, leading, in turn, to a loss of the VWF tubular structures, and thus a loss of the cigar shape). This could allow endothelial cells to tune their response to different secretagogues, allowing a release of content that has a lower ratio of VWF to cytokines and is thus less thrombotic and more inflammatory.

Thus, the overall mechanism by which WPBs engage with the plasma membrane to release their content is still not completely understood; and what of the molecular machinery that will drive this multistep process?

### Tethering, docking and fusion machinery

The first cellular machinery to be identified as likely to be involved in tethering WPBs at the plasma membrane was identified by Voorberg *et al*. They found the small GTPase RalA to be associated with WPBs 46, and went on to show that expression of mutants of this protein could strongly affect the evoked release of VWF. RalA is a component of the exocyst, a tethering complex that also interacts with soluble *N*-ethylmaleimide-sensitive factor (NSF) attachment protein receptors (SNAREs) and their regulators, including Rabs – see below. In addition to tethering factors, Munc13-4 47 and Munc18c 48, a member of another family of SNARE modulators – the SM proteins – have been identified as playing a role in VWF secretion. SM proteins can regulate exocytosis by binding to syntaxins (plasma membrane Q-SNAREs) and modulating their conformation 49. It is noteworthy that Munc13-4 was confirmed to be expressed in HUVECs by Zografou *et al.* 47, and colocalizes with WPBs. It has also been shown to act as an effector of Rab27A (see below). Yet another regulator of fusion involved in endothelial exocytosis is Slp4a/granuphilin. Granuphilin is a positive regulator of VWF secretion 50 and is a third effector of Rab27A. It has been shown to act in docking insulin granules to the plasma membrane in pancreatic β-cells 51.

It is noteworthy that Rab27A and (particularly) granuphilin are located at the very tip of the WPB that then fuses with the plasma membrane (Nightingale and Cutler, unpublished data). Rab27a is thus involved in more than one stage of secretion, acting in different ways on each occasion. How this change in function is managed is not clear, although it must be centered on a change in effector.

Finally, the annexin A2–S100A10 complex has also been shown to be needed for VWF secretion 52, but, although it probably acts late in exocytosis at the plasma membrane (possibly playing a regulatory role via its lipid-binding and calcium-binding properties), just how it fits into the process is not yet clear.

In attempting to unravel some of the complexities of VWF secretion, Bierings *et al.* 50 carried out a comparative analysis of both MyRIP and Slp4a. Slp4a can bind plasma membrane-associated syntaxins 53,54 and Munc18 55–57, to link secretory granules to exocytic SNAREs. It can also interact with Rabs other than Rab27A, including Rab3A, Rab3B, Rab3C, and Rab3D 58, and with MyoVa 59. They found that MyRIP is a negative regulator of VWF release in response to histamine, in agreement with Nightingale *et al*., whereas Slp4a acts as a positive regulator. This suggests that Slp4a interacts with the exocytic machinery in the endothelial system, again suggesting that there may be a population of WPBs within HUVECs that are somehow docked at the plasma membrane, or a role for a docking phase distinct from the earlier anchoring phase. They conclude by proposing that the balance of the two effectors recruited by Rab27A will determine the probability of release of WPBs 50.

The other Rab that is associated with WPBs is Rab3D 52, and overexpression of mutants of this Rab has been reported to interfere with WPB formation and thereby affect VWF secretion. However, Bierings *et al*. reported that siRNA suppression of Rab3B or Rab3D interferes with the recruitment of Slp4a but not with VWF secretion; they found that exocytosis was affected only via suppression of Rab27A. They did, however, confirm the inhibitory effect of overexpression of Rab3D on secretion, but found that this was indirect, and resulted from competition of Rab27a for Slp4a effector proteins. They also mention that Rab3B is the major isoform, with 95% of expression, with Rab3D representing only 5% of expressed Rab3 50.

A set of data from Zografou *et al.* 47 significantly complicates this picture. They carried out an overexpression screen of green fluorescent protein-tagged Rabs in HUVECs, and found not only those reported by others – Rab27A, Rab3B, and Rab3D – but also that the other isoforms of Rab3, the other isoform of Rab27, as well as Rab15, Rab33A, and Rab37, are targeted to WPBs. Of these, Rab15 is the most unexpected, as an established endocytic Rab. They depleted these Rabs by siRNA ablation, and, by examining their role in secretion with a mixture of ATP, vascular endothelial growth factor and basic fibroblast growth factor as secretagogues, determined that Rab3A, Rab3D, Rab27A and Rab15 are all needed for secretion of VWF. Thus, while confirming the role of Rab27A, they introduce a further three Rabs as being required for exocytosis of WPBs. Rab3A was not found to be expressed in HUVECs by Bierings *et al*., although overexpression of this isoform showed it to localize to WPBs in HUVECs. Both Knop and Bierings have demonstrated that Rab3D is expressed in HUVECs and colocalizes with WPBs, but Bierings and Zografou disagree as to whether it functions in exocytosis. In overexpression studies, Rab3D was reported by Knop to affect the formation of WPBs, by Bierings, using siRNA ablation, not to affect the release of VWF, but by Zografou, again using siRNA, to indeed affect the release of VWF. Thus far, only Zografou *et al*. have reported a WPB localization and function for Rab15.

Zografou also introduce analyses of the third Rab effector to be studied in endothelial cells, Munc13-4 47. This protein, identified as a Rab27A effector that is widely expressed in hemopoietic cells, is involved in the priming of secretory granules, and promotes SNARE complex formation 60. Zografou *et al*. have found that siRNA suppression of Munc13-4 reduces VWF secretion and that, in addition to Rab27A, it interacts with Rab15. They suggest that both Rabs act through this effector. The role of an endocytic Rab (15) in exocytosis of WPBs is difficult to interpret, but they suggest that it might relate to the well-known delivery from endosomes of WPB components, including CD63, the essential cofactor of the WPB integral membrane protein P-selectin 61.

The studies by Bierings *et al*. and Zografou *et al*. found a fall in the evoked release of VWF from Rab27A-depleted cells; this is in contrast to the increase in release demonstrated by Nightingale *et al*. Bierings *et al*. did, however, confirm the negative regulatory role for the Rab27a effector MyRIP suggested by Nightingale *et al*. The difference in Rab27a depletion phenotype between these two studies may reflect the different timescales of their depletion experiments. If MyRIP and Rab27a do, as hypothesized, provide a brake that allows maturation, then longer-term depletion of Rab27A could result in an entirely immature population of WPBs that are hyperresposive to secretagogue. Such an effect may be masked by the other Rab27a effectors (Munc13-4 and Slp4a) in a shorter experimental timescale.

Many of these proteins are probably operating on the fusion machinery at the heart of exocytosis. The core components of this fusion machinery are the SNARE complexes, and for WPBs in endothelial cells these are currently thought to be Vamp3, syntaxin 4, and SNAP23. Lowenstein *et al.* 62 first suggested that Vamp3 on the WPBs, and syntaxin 4 and SNAP23 on the plasma membrane, were the SNARE complexes involved in WPB fusion in experiments exploring how nitric oxide inhibits vascular inflammation. After identifying NSF nitrosylation as the cause of the reduced inflammatory response, they showed that antibodies against VAMP3 and Syt4 reduced the secretion of VWF from permeabilized human aortic endothelial cells. Fu *et al.* 48 followed this by showing that Syt4 was phosphorylated following endothelial activation, and that its suppression by siRNA reduced P-selectin exposure at the plasma membrane of activated human lung microvascular endothelial cells. More recently, Pulido *et al.* 63 performed a focused study of WPB SNAREs in HUVECs, using combinations of permeabilized cells, siRNA ablation, and mutant proteins, and confirmed that Vamp3, Syt4 and SNAP23 are likely to be the core components of the WPB exocytic SNARE complex.

To further complicate analyses of VWF secretion with another layer of regulatory machinery, Nightingale *et al.* 44,^64^ have described yet another way in which endothelial cells can modulate the secretory consequences of fusion. They described a novel postfusion role for actin in VWF secretion. In addition to F-actin providing a structure for Rab27A to anchor WPBs via MyRIP and MyoVa (see above), thus facilitating their maturation, they found that an actomyosin IIB ring or cup is rapidly formed around the plasma membrane-distal end of the WPB immediately after fusion, and that this is needed for extrusion of VWF: Poisoning of myosin IIB allows the fused WPB to remain fused at the plasma membrane with its cargo of VWF sitting in the collapsed WPB.

Thus, mechanisms involved in tethering, docking, priming, fusion (albeit only as defined in other secretory systems) and cargo extrusion have been found, but these are only the bare bones of the exocytic process for WPB; how they interact, what other components are involved and how they interact with the signaling pathways arising on endothelial activation (and which have been reviewed elsewhere) are still to be established.

One interesting new way of finding new components may be through genome-wide association studies of VWF and FVIII plasma levels and the associated risk of thrombosis 65. Recent analyses of this type have found that not only a syntaxin-binding protein (syntaxin-binding protein 5) but also another syntaxin (syntaxin 2) have been associated with increases in the levels of plasma VWF and thrombosis 66–69. The former protein is also known as tomosyn, and is thought to regulate exocytosis in other systems 70,71, whereas the latter has not previously been thought to be involved in VWF secretion. Whether components identified by broad genetic analyses are directly involved in WPB exocytosis, or act more indirectly, remains to be established. Indeed, whether a great deal of further machinery is still to be found is unclear; certainly, there are significant issues still unresolved.

One puzzle relates to the docking of WPBs in endothelial cells. A conventional view of granule docking, mainly derived from neuroendocrine granules, has developed from a morphologic definition arising from the secretory organelles being closely apposed to the plasma membrane. In endothelial cells, this has not yet been established, although the machinery for doing so may be emerging, but ‘docking’ by anchoring to filamentous actin has been described. Whether both of these processes occur separately in the endothelium, and, if so, how they relate to each other is as yet unclear.

## Conclusions

As should be clear from the above discussion, the identity and functioning of Rabs associated with WPBs has been a particularly active area of WPB research in the last few years. This is because the small GTPases of the Rab family act as master controllers of cellular membrane biology, acting from organelle formation to exocytosis 72, Thus far, Rabs identified as localizing to WPBs have only been found on mature organelles. These include Rab3A, Rab3B, Rab3C, Rab3D, Rab15, Rab27A, Rab27B, Rab33A, and Rab37 26,34,47,50,52. The other Rabs playing a role in VWF secretion are Rab10 and Rab8A, which do not colocalize with WPBs but are associated with the Golgi in HUVECs, and were found to interact genetically with AP-1 (in a *Caenorhabditis elegans* screen) and therefore possibly play a role in WPB biogenesis 73. It is interesting that Rab8 has also been shown to interact with the exocyst, and is probably involved in exocytosis (see above), but is also thought to act in trafficking from the Golgi 74.

In addition, each Rab will act through its effectors, recruited by the Rab in its GTP-loaded state, when it is switched on. The list of investigated endothelial WPB-associated effectors (MyRIP, Slp4a, and Munc13-4) is therefore currently shorter than the numbers of Rabs, and some are known to interact with more than one of the identified Rabs. This very large collection of Rabs is consistent with their playing more than one role in WPB functioning and VWF secretion, which will make discovering precisely what might be their individual contribution, within a system of cooperation and competition, extremely challenging. As discussed above, the picture is further complicated by discrepancies between the published reports.

A necessarily speculative summary of the current Rab-related data would place Rab27 at the center of the control of exocytosis. It acts to anchor WPBs to actin via MyRIP and MyoVa to support maturation, and then switches to other effectors to allow it to bind to the SNARE complex at the plasma membrane via granuphilin and/or Munc13-4 to support fusion. Rab3D and Rab3B might assist in recruiting some effectors, and Rab15 could play a role in reinforcing the later SNARE-binding stages of exocytosis. In other organellar systems where multiple Rabs are present on a single organelle, a Rab cascade can occur, whereby an upstream Rab recruits the GDP/GTP exchange factor for the downstream Rab; this cascade can be further bolstered by the downstream Rab subsequently recruiting a GTPase-activating protein specific for the upstream Rab. This is best understood for Rab5 and Rab7 75–77, and has been hypothesized for Rab9 with Rab32 and Rab38 78. Such a cascade has been suggested for other regulated storage organelles that recruit both Rab27a and Rab3, such as the acrosome 79. Where active Rab27a increases the likelihood of the presence of GTP-loaded Rab3, such a situation could allow the handover of common effector proteins. This does not, however, fit with the release of WPBs; first, Rab3 can be found on Rab27a-deficient WPBs, and second, removal of Rab27a and of Rab3 give different secretory phenotypes. As yet, it is unclear what could allow the balance of Rab27a effectors to shift from MyRIP to Slp4a or Munc13-4 (i.e. from negative to positive effector). Perhaps, as suggested by Bierings *et al*., it could result from the fact that Slp4a can bind GDP-bound Rab, or from the increased affinity of Slp4a as compared to that of MyRIP for Rab27a. This will provide an interesting and active area of research.

In general, much of the data now available suggest that differences between experimental conditions may affect the outcome of experiments, and that interpretation of such an overall picture will require caution and further extensive analyses. Altogether, the state of play with respect to VWF secretion is that many of the stages are being defined, both morphologically and by molecular mechanistic analyses, but that such studies are early stages in the task of producing a fully integrated and detailed picture of how such a complex process acts in an integrated and physiologically responsive way. What can be said at this early stage is that the secretion of VWF is likely to occur via one of the most highly regulated secretory systems, and that the interest in this is unlikely to disappear anytime soon.

## Disclosure of Conflict of Interests

The authors state that they have no conflict of interest.
